# Treatment and care of TB across Europe

**DOI:** 10.7448/IAS.17.4.19503

**Published:** 2014-11-02

**Authors:** Ole Kirk

**Affiliations:** Copenhagen HIV programme, University of Copenhagen, Copenhagen, Denmark

## Abstract

There continues to be profound differences in TB incidences as well as in TB treatment and care across Europe in recent years. High TB incidences are observed in Eastern Europe and especially among injecting drug users (IDUs), and there are large overlaps with a HIV epidemic, which is also far from being under control in this region. Further, mortality rates among HIV-positive patients with TB in Eastern Europe were in the 2000s 3–5 fold higher compared with other parts of Europe. As of 2014, there remain many challenges in the organizational set-up of TB care in Eastern Europe and often only limited possibilities of support for the vulnerable IDU populations, including in particular opioid substitution therapy. Further, high prevalences of multi-drug-resistant TB (MR-TB) are an increasing problem in Eastern Europe which in several settings reached beyond 50% among patients previously treated for TB. Obviously, this causes problems for immediate therapy of the individual patients and for development of secondary resistance during therapy. The panel of TB drugs available in TB clinics in Eastern Europe is often limited, and a quick TB diagnosis and acknowledgement of the resistance pattern to guide initial therapy seems to be a priority. For HIV-positive patients in particular, better access to cART may also be an important tool to reduce the TB incidence among these patients. Thus, treatment and care of TB patients in especially Eastern Europe are facing a variety of diagnostic and therapeutic challenges in the coming years.

